# The Care and Control of the Feeble-Minded

**Published:** 1908-08-08

**Authors:** 


### The Hospital

A Journal of

The Medical Sciences and Hospital Administration.

New Series. No. 74, Vol. III. [No. 1146, Vol. XLIV.] SATURDAY, AUGUST 8, 1908.

### THE CARE AND CONTROL OF THE FEEBLE-MINDED.

Almost four years after its formation the Royal Commission on the Care and Control of the Feeble-minded has issued its report in a volume of 470 pages. Minutes of evidence, appendices, reports on medical investigations and on visits to America, which will be published this month, are to fill seven other volumes, from which the magnitude of the Commission's task will be appreciated. It is believed that there are in England and Wales 149,628 mentally defective persons, or .46 per cent. of the population, compared with 271,607, or .83 per cent., of certified lunatics. For purposes of definition nine varieties are recognised, the first including those who are commonly termed insane: the other eight are the mentally infirm, who through age or other cause of decay are incapable of managing their affairs; idiots, who require constant supervision in their own interests; imbeciles, similarly but less severely affected; the feeble-minded, who do not compete on level terms with their fellows; moral imbeciles, including criminal degenerates; epileptics, inebriates, and the deaf, dumb, or blind, who are also mentally defective. The Commission conclude that with regard to all those who can be so classed the present system is wasteful and inefficient, besides allowing much unnecessary crime and misery. The permissive education which local authorities may provide for such children is "supplemented by no subsequent supervision and control, and is in consequence often misdirected and unserviceable." The Commission finds lunatic asylums full of many who could be just as well, and far more economically, looked after at home; and at the same time numbers at large who are exposed to constant moral danger to themselves and the source of lasting injury to the community.

Proceeding to the remedies for this unsatisfactory state of things, the Commission points out that at present there is no settled plan of action and co-operation between the various authorities—such as the Poor-law, schools, prisons, etc.—under whose care the feeble-minded may come, and that some supervising authority is required to co-ordinate the work. Inasmuch as some of the most important changes suggested will require legislation, it is evident that nothing can be usefully attempted in this direction until the conclusions of the two kindred Commissions on the Poor-law and the Inebriates Acts have been made public. But for certain immediate purposes and pending legislation, the Lord Chancellor has certain powers which it is recommended he should exercise. Thus he can order the amalgamation of the office of the Chancery Visitors and their duties and staff with those of the Lunacy Commission, and he can add two medical men to the latter. The first of these reforms is pressed for by the Royal Commission as a matter of great urgency.

More important, however, than these stop-gap measures is the recommendation that a Board of Control should be constituted for the general protection and supervision of the mentally defective, and for the regulation of their entire education, treatment, and control. The proposed Board is to be composed of alienists and barristers, in proportions not definitely laid down as yet. It is pointed out how much more extensive than those of the Lunacy Commission will be the powers and functions of this Board, and a somewhat significant paragraph is added expressing the hope that the selection of its members will be governed entirely by suitability, and "be biassed by no other consideration whatever." In that sentence, which is one of the most remarkable ever inserted in the report of a Royal Commission, is recognised the rock on which the entire scheme may split. It is indeed hopeless to expect any useful result from the establishment of a Board of Control if it is to be exploited by providing thereon well-paid posts for political hacks, and the more plainly that is stated at the outset the better it will be for all concerned. The County and Borough Councils are to undertake the care of the feeble-minded in their populations, and are to be responsible for this to the Board of Control; and as this recommendation applies to London as well as the rest of the country, the County Council will be expected to replace the existing Asylums Committee by a new statutory committee, which will control also the education of, and all public and voluntary provision for, the feeble-minded in the Metropolis.

There are many other most interesting points dealt with in this exhaustive report. To the medical profession the conclusions on heredity are instructive. It is held that "feeble-mindedness" is usually spontaneous in origin—that is not due to influences acting on the parent—and tends strongly to be inherited; and that in view of the evidence concerning fertility, the prevention of the mentally defective from becoming parents would tend largely to diminish the number of such persons in the population. Now the first of these statements is obscure and almost self-contradictory; it seems to mean that alcoholism, syphilis, and other factors commonly credited with the causation of degeneracy in the next generation have no such effect, which is a most remarkable conclusion, and one for which the evidence, when published, will be carefully scrutinised. The second statement will command more general assent, though we are sorry to see that the Commissioners refuse to push it to its logical conclusion. They say that in their opinion the general feeling of the people would at present rightly condemn any legislation directed to this end, and that only three witnesses out of twenty-one thought it practicable. To make the sentimentality of the uninstructed public an excuse for shirking an unpopular but beneficial recommendation is just one of the things that a non-party Royal Commission ought to be superior to. Such a very elementary application of the principles of eugenics must come in time, and in refusing to entertain it the Commissioners out of their own mouths stand convicted of weak-kneed vacillation. Since the discovery that azoospermia without loss of sexual power can be produced by exposure to $x$-rays, there is less reason than ever for declining to protect society from its drones and parasites by the application of that very simple and unobjectionable method of sterilisation.

